# Estrogen receptor-dependent genomic expression profiles in breast cancer cells in response to fatty acids

**DOI:** 10.4103/1477-3163.59539

**Published:** 2010-02-04

**Authors:** Faizeh Alquobaili, Stacy-Ann Miller, Seid Muhie, Agnes Day, Marti Jett, Rasha Hammamieh

**Affiliations:** School of Pharmacy, Damascus University, Syria, USA; 1 Department of Molecular Pathology, Walter Reed Army Institute of Research, Silver Spring, USA; 2 Department of Microbiology, Howard University, Washington DC, USA

**Keywords:** Estrogen receptor, breast cancer, microarray, fatty acids, omega-3, omega-6

## Abstract

**Context:**

The estrogen receptor (ER) status in breast cancer plays a major role in the progression and metastatic potential of breast cancer in women. Breast cancer cells lacking the ER are usually more advanced and more difficult to treat than ER+ breast cancer cells. ER− women have more advanced breast cancer at the time of diagnosis than ER+ women. ER− breast cancer cells in women, regardless of age, are more likely to have tumor Grade III or IV with fewer Grade I and II tumor stages combined for each individual stage group. Studies have suggested a strong correlation between fat intake and the elevated risk of ER+ breast cancer cells.

**Materials and Methods:**

We studied the role of ER status on the gene expression in breast cancer cells in response to omega-3 and omega-6 fatty acids using microarrays. We have studied gene expression patterns in 8 breast cancer cell lines (4 ER− and 4 ER+) in response to Eicosapentanoic (EPA) and Arachidonic (AA) acids.

**Statistical Analysis:**

Analysis of Variance (ANOVA) t-test analysis was carried out to identify genes differentially expressed between the two groups.

**Results:**

We identified genes which were significantly correlated with the ER status when breast cancer cells were treated with these fatty acids.

**Conclusion:**

We have determined ER-related gene expression patterns in breast cancer cells in response to fatty acids. Additional studies of these biomarkers may enlighten the importance of the ER status on the mechanistic and therapeutic roles of fatty acids in breast cancer.

## INTRODUCTION

Estrogens control the growth and differentiation of mammary glands and regulate gene expression in breast cells through the estrogen receptor (ER). ERs are expressed in 70% of breast cancer cases where cancer cell growth is controlled by estrogen and is often susceptible to treatment with inhibitors that block the interaction between estrogen and the estrogen receptor.

The estrogen receptor status in breast cancer plays a major role in the progression and metastatic potential of breast cancer in women. Breast cancer cells lacking the (ER−) are usually more advanced and more difficult to treat than ER+ breast cancer cells. A disparity in breast carcinoma survival between ER− and ER+ cases has been noted over the past several decades. ER− women have more advanced breast cancer at the time of diagnosis than ER+ women. In addition, ER− women tend to have breast cancer tumor types that are more aggressive and have poorer prognosis. ER− breast cancer cells in women, regardless of age, are more likely to have tumor grade III or IV with fewer grade I and II tumor stages combined and for each individual stage group.

Epidemiologic studies have found a significant correlation between ER+ breast cancer cells and several lifestyle risk factors, such as higher body mass index, earlier age at menarche, nulliparity, and diet.[1–3] Cho *et al.* studied the association between dietary fat intake and breast cancer in premenopausal women and found a strong correlation between fat intake and the elevated risk of ER+ breast cancers.[4]

A case-case study that evaluated the association of dietary fat intake of selected fatty acids found that high intakes of linoleic acid in premenopausal breast cancer patients were associated with a threefold higher risk of ER− than ER+ tumors.[5]

The disparities observed in incidence trends and age at diagnosis highlight the need for further investigation of the differences between ER− and ER+ breast cancer cells. Gruvberger *et al.* studied gene expression profiles in ER− and ER+ breast tumors using microarrays and showed that they had very distinct gene expression patterns.[6] The study found a significant increase in the expression levels of *P-cadherin*, *C/EBP* β transcription factor, and *ladinin* in ER− breast cancer cells. It also identified *GATA3*, *Cyclin D1* and *carbonic anhydrase XII* expression to be associated with ER+ breast cancer samples.

In a previous study, we characterized the transcriptional profiles in breast cancer cells treated with omega-3 and omega-6 fatty acids.[7] In that study, we observed differences in gene expression between ER+ and ER− cells in response to the fatty acids, but this was a preliminary finding since only 2 cell lines of each ER status were used; therefore we doubled the number of each group in order to identify gene expression profiles directly associated with ER status. We are now able to describe in more detail the role of ER status on the gene expression in breast cancer cells in response to omega-3 and omega-6 fatty acids using the 4 well-characterized ER− and 4 ER+ breast cancer cells. We identified the genes that were significantly correlated with the ER status when breast cancer cells were treated with these fatty acids.

Note: microarray data have been submitted to the Gene Expression Omnibus (GEO) and can be searched using the Platform ID: GPL8144, Series: GSE14679.

## MATERIALS AND METHODS

ER− (HCC-1806, MDA-MB-468, Hs578T and SK-BR-3) and ER+ (HCC-70, MCF-7, HCC-1500 and CAMA-1) breast cancer cell lines as well as culture media were obtained from ATCC (Manassass, VA). Fatty acids were obtained from BioMol (Plymouth Meeting, PA). Each fatty acid was aliquoted and aliquots were stored at -70°C until used. The TRIzol™ reagent was obtained from Invitrogen (Carlsbad, CA), iScript cDNA synthesis kit from Bio-Rad (Hercules, CA) and the Micromax Tyramide Signal Amplification (TSA) and Labeling Kit from Perkin Elmer, Inc. (Wellesley, MA).

Cell lines were cultured in the recommended media. Twenty four hours prior to treatment with fatty acids, culture media were removed and cells were washed with PBS and incubated in the same media supplemented with 1% (v/v) insulin/sodium selenite and 1% (v/v) non-essential amino acids in the absence of FBS. At the scheduled times, selected flasks were treated with 10 *μ*M fatty acids added to fresh media and incubated for six and 24 hours respectively. Control cells were incubated in fresh media in the absence of fatty acids.

Total RNA was isolated using TRIzol reagent (Invitrogen, CA) following the manufacturer's protocol. RNA quality and quantity were determined on an Agilent 2100 Bioanalyzer (Agilent Technologies, CA).

Human cDNA microarrays were prepared as described in Hammamieh *et al.*[8] Briefly, we used sequence verified oligos (∼36,000 oligos) representing the whole genome (Operon, Inc, Huntsville, AL). The oligos were deposited in 3X saline sodium citrate (SSC) at an average concentration of 165 βg/ml on CMT-GAPS II aminopropyl silane-coated slides (Corning, NY), using a VersArray microarrayer (Bio-Rad, Inc). Arrays were post processed using UV-cross linking at 1200 mJ/cm^2^ and by baking for four hours at 80°C. Positively charged amine groups (on the slide surface) were then treated with succinic anhydride and N-methyl-2-pyrrolidinone.

Microarray slides were labeled using Micromax Tyramide Signal Amplification (TSA) Labeling and Detection Kit (Perkin Elmer, Inc., MA) as described in Hammamieh *et al*.[7] Slides were hybridized for 16 hours at 60 °C. Hybridized slides were scanned using GenePix Pro 4000B optical scanner (Axon Instruments, Inc., CA). Intensities of the scanned images were digitalized through Genepix 6.0 software.

Assessment of the overall integrity of the microarray experiment were carried out as described in Hammamieh *et al*:[8]

Microarray images were visualized and normalized using ImaGene 6.0 (BioDiscovery, Inc., CA); and data was analyzed using GeneSpring 10.1 (Agilent Technologies, CA).

Background and foreground pixels of each spot were segmented using ImaGene (BioDiscovery Inc., CA), and the highest and lowest 20% groups of the probe intensity were discarded. Local background correction was applied to each individual spot. The genes that passed this filter in all the experiments were further analyzed.

Data filter and statistical analysis were carried out using GeneSpring 10.1. Local background was subtracted from individual spot intensity and genes that failed ‘background check’ in any of the experiments were eliminated from further analysis. Each chip was next subjected to intra-chip normalization (LOWESS). Differentially regulated genes (between control and treated sample sets) were selected using *t*-test analysis (P < 0.05).

Principal component analysis (PCA) was performed over the given dataset, classifying each sample as a statistical variable in order to confirm the extent of variability within the sample classes and among the pre-designed groups. A two-dimensional hierarchal clustering calculation using Pearson correlation around zero was also performed.

We randomly selected genes to confirm their expression profiles using real time PCR. These genes are *Protocadherin, thyroid hormone receptor-associated protein complex component (TRAP150), Mitochondrial ribosomal protein L43, transducer of ERBB2, WNT-2B Isoform 1 oncogene and coiled-coil domain containing 61 (CCDC61).* Primer3, A web-based primer designing tool, was used to design primers for selected genes (http://www.frodo.wi.mit.edu/). The specificity of each primer sequence was confirmed by running a blast search. Reverse transcription and Real-time PCR reactions were carried out using iScript cDNA synthesis kit from Bio-Rad (Hercules, CA) and a Real-time PCR kit (Roche, IN), respectively. Each reaction was run in I-Cycler (Bio-Rad, CA) using five technical duplicates. Each sample was also amplified using primer sets for the 18S house-keeping probe of the experiment. The resultant cycle threshold data from each real-time-PCR ‘run’ was converted to fold-change.

## RESULTS

We have studied gene expression profiles and identified genes differentially expressed between ER− and ER+ breast cancer cells treated with EPA.

**Figure 1:**
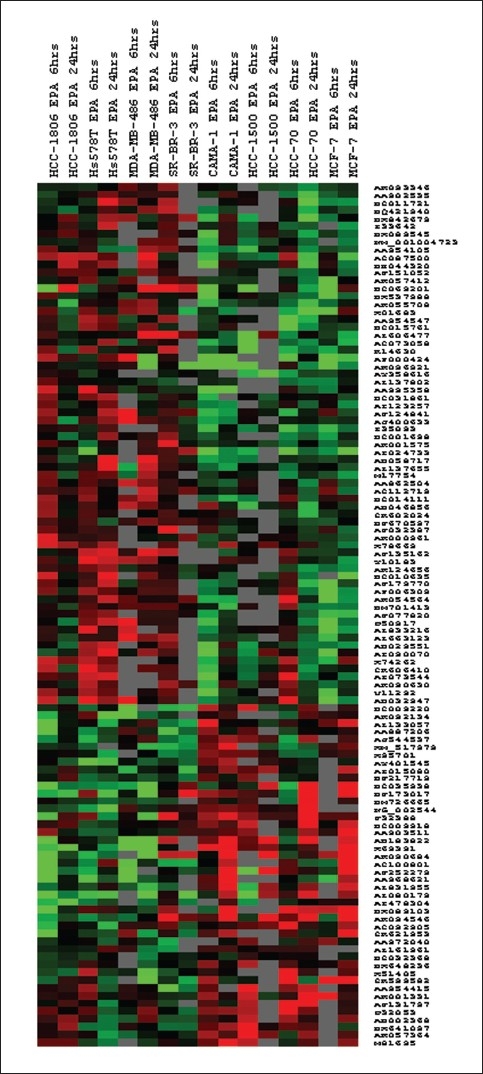
A pseudo color cluster view of genes differentially expressed between ER− and ER+ breast cancer cells in response to EPA. Cells were treated with EPA at 6 and 24 hrs. RNA was isolated and hybridized on the cDNA microarray slides as detailed in materials and methods. Images were analyzed using GenePix 6.0 and data were analyzed using GeneSpring 10.1

Data were normalized by applying inter-chip and intra-chip normalizations using GeneSpring 10.1, as described in the methods section. When we used One-way ANOVA with a P-value < 0.05 we identified 819 genes, out of the 36000 genes, to be differentially expressed between ER− and ER+ breast cancer cells in response to treatment with EPA [Figure 1].

To functionally classify the genes associated with the ER status in breast cancer cells treated with EPA, we used GeneSpring 10.1 and FATIGO+.[9,10] We have also used Ingenuity Pathway Analysis and GeneCite to carry out detailed pathway analysis using the Biocarta pathways.[11] Functional classification of up regulated genes revealed that genes involved in the G2/M DNA damage checkpoint regulation, protein ubiquitination and apoptosis signaling were up regulated in ER+ cells in response to EPA while the cyclin dependent kinase signaling cascade was associated with ER− cells.

**Figure 2:**
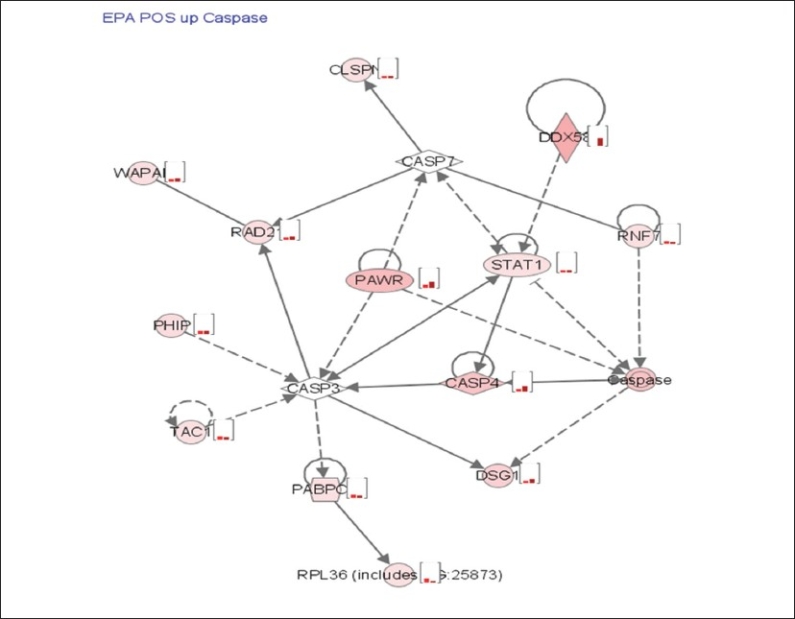
Ingenuity pathway analysis and expression profiles of genes involved in apoptosis that were uniquely up regulated in ER+ cells in response to EPA. Cells were incubated with EPA for 6 and 24 hrs. RNA samples were isolated and hybridized on the cDNA microarray slides as detailed in materials and methods. Images were analyzed using GenePix 6.0 and data were analyzed using GeneSpring 10.1

Ingenuity analysis of the genes up regulated in ER+ cells identified an apoptosis related network as being significantly enriched and among the top ranked networks. Some of these genes included caspases and STAT1. They are listed in Table 1 and a simplified network is depicted in Figure 2. Hypoxia-inducible factor 1 (HIF1-α), hypoxia inducible factor 3(HIF3-α) DEAD (Asp-Glu-Ala-Asp) box polypeptide 21, CHK1 checkpoint homolog, cyclin-dependent kinase inhibitor 2A (CDKN2A) and the CDKN2A interacting protein (CDKN2AIP) were also uniquely up regulated in ER+ cells [Table 2].

**Table 1:** Apoptosis related genes up regulated in ER+ breast cancer cells in response to EPA

Symbol	Entrez gene name	Fold change
CASP4	caspase 4, apoptosis-related cysteine peptidase	3.5
CLSPN	claspin homolog (Xenopus laevis)	1.8
DDX58	DEAD (Asp-Glu-Ala-Asp) box polypeptide 58	5.0
DSG1	desmoglein 1	2.7
PABPC1	poly(A) binding protein, cytoplasmic 1	1.5
PAWR	PRKC, apoptosis, WT1, regulator	4.0
PHIP	pleckstrin homology domain interacting protein	2.1
RAD21	RAD21 homolog (S. pombe)	2.0
RNF7	ring finger protein 7	1.3
RPL36	ribosomal protein L36	1.2
STAT1	signal transducer and activator of transcription 1, 91kDa	1.5
TAC1	tachykinin, precursor 1	1.6
WAPAL	wings apart-like homolog (Drosophila)	1.9

**Table 2:** HIF Pathway related genes up regulated in ER+ breast cancer cells in response to EPA

Symbol	Entrez gene name	Fold change
ARNT	aryl hydrocarbon receptor nuclear translocator	2.0
SFRS1	splicing factor, arginine/serine-rich 1	1.2
DDX3X	DEAD (Asp-Glu-Ala-Asp) box polypeptide 3, X-linked	1.5
RPL8	ribosomal protein L8	1.5
HNRNPM	heterogeneous nuclear ribonucleoprotein M	1.5
HNRNPA2B1	heterogeneous nuclear ribonucleoprotein A2/B1	1.5
DACH1	dachshund homolog 1 (Drosophila)	1.6
HIF1A	hypoxia-inducible factor 1, alpha subunit	1.7
NRN1	neuritin 1	1.7
HDGF	hepatoma-derived growth factor (high-mobility group protein 1-like)	1.8
CDKN2A	cyclin-dependent kinase inhibitor 2A (melanoma, p16, inhibits CDK4)	1.8
CLSPN	claspin homolog (Xenopus laevis)	1.8
RBM39	RNA binding motif protein 39	1.8
NUP50	nucleoporin 50kDa	1.8
HIF3A	hypoxia inducible factor 3, alpha subunit	1.9
CDKN2AIP	CDKN2A interacting protein	2.1
DDX21	DEAD (Asp-Glu-Ala-Asp) box polypeptide 21	2.2
CHEK1	CHK1 checkpoint homolog (S. pombe)	2.4
DSG1	desmoglein 1	2.7
NPM1	nucleophosmin (nucleolar phosphoprotein B23, numatrin)	2.8

Our data show that genes involved in cell-to-cell signaling were up regulated only in ER− cells when treated with EPA. These genes include the Cyclin-dependent kinase 4 (CDK4), cell division cycle 7 (CDC7), cyclin-dependent kinase 4, fragile X mental retardation 1, protein phosphatase 1 catalytic subunit beta isoform (PPP1CB), protein phosphatase 1 regulatory (inhibitor) subunit 12A (PPP1R12A), protein phosphatase 2 catalytic subunit alpha isoform (PPP2CA) and protein phosphatase 2 regulatory subunit A alpha isoform (PPP2R1A).

**Figure 3:**
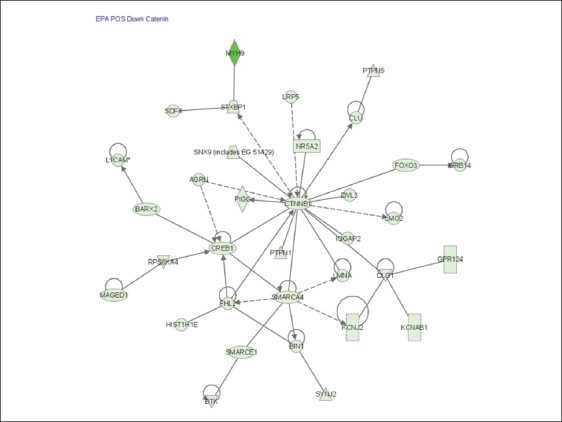
β-catenin cascade that was uniquely down regulated in ER+ cells in response to EPA. Cells were incubated with EPA for 6 and 24 hrs. RNA samples were isolated and hybridized on the cDNA microarray slides as detailed in materials and methods. Images were analyzed using GenePix 6.0 and data were analyzed using GeneSpring 10.1

Among the genes that were down regulated in ER+ cells in response to EPA were genes involved in β-catenin signaling and the BCL-2 anti-apoptosis pathway [Figure 3]. Table 3 lists the name and the regulation patterns of genes involved in the β-catenin pathway.

**Table 3:** Beta-catenin signaling and the BCL-2 antiapoptosis pathway genes down regulated in ER+ cells in response to EPA

Symbol	Entrez Gene Name	Fold Change
AGRN	agrin	−2.0
BARX2	BARX homeobox 2	−1.6
BIN1	bridging integrator 1	−2.4
BTK	Bruton agammaglobulinemia tyrosine kinase	−2.6
CLU	clusterin	−2.4
CREB1	cAMP responsive element binding protein 1	−1.1
CTNNB1	catenin (cadherin-associated protein), beta 1, 88kDa	−1.5
DLG1	discs, large homolog 1 (Drosophila)	−1.5
DVL3	dishevelled, dsh homolog 3 (Drosophila)	−3.7
FHL2	four and a half LIM domains 2	−2.1
FOXO3	forkhead box O3	−2.3
GPR124	G protein-coupled receptor 124	−1.5
GRB14	growth factor receptor-bound protein 14	−1.4
HIST1H1E	histone cluster 1, H1e	−1.6
IQGAP2	IQ motif containing GTPase activating protein 2	−5.2
KCNAB1	potassium voltage-gated channel, shakerrelated subfamily, beta member 1	−1.7
KCNJ2	potassium inwardly-rectifying channel, subfamily J, member 2	−2.1
L1CAM	L1 cell adhesion molecule	−10.6
LMNA	lamin A/C	−1.1
LMO2	LIM domain only 2 (rhombotin-like 1)	−1.6
LRP5	low density lipoprotein receptor-related protein 5	−2.0
MAGED1	melanoma antigen family D, 1	−1.6
MYH9	myosin, heavy chain 9, non-muscle	−100.0
NR5A2	nuclear receptor subfamily 5, group A, member 2	−1.2
PIGC	phosphatidylinositol glycan anchor biosynthesis, class C	−1.5
PTPN1	protein tyrosine phosphatase, non-receptor type 1	−1.7
PTPN5	protein tyrosine phosphatase, non-receptor type 5 (striatum-enriched)	−1.9
RPS6KA4	ribosomal protein S6 kinase, 90kDa, polypeptide 4	−1.6
SDF4	stromal cell derived factor 4	−2.4
SMARCA4	SWI/SNF related, actin dependent regulator of chromatin, subfamily a, member 4	−2.9
SMARCE1	SWI/SNF related, actin dependent regulator of chromatin, subfamily e, member 1	−1.0
SNX9	sorting nexin 9	−3.8
STXBP1	syntaxin binding protein 1	−1.4
SYNJ2	synaptojanin 2	−1.2

The genes of amino acid synthesis pathway were highly enriched in the list of genes down regulated by EPA in ER− cells. These genes are listed in Table 4. We have studied gene expression profiles and identified genes differentially expressed between ER− and ER+ breast cancer cells treated with AA.

**Table 4:** Amino acid synthesis related genes down regulated in ER- cells in response to EPA

Symbol	Entrez Gene Name	Fold Change
BMPR1B	bone morphogenetic protein receptor, type IB	−1.3
CYP1A2	cytochrome P450, family 1, subfamily A, polypeptide 2	−8.3
DR1	down-regulator of transcription 1, TBPbinding (negative cofactor 2)	−1.4
ECOP	EGFR-coamplified and overexpressed protein	−1.6
GRIN3A	glutamate receptor, ionotropic, N-methyl-Daspartate 3A	−1.7
HK1	hexokinase 1	−1.5
HGFAC	HGF activator	−4.5
IHPK1	inositol hexaphosphate kinase 1	−1.8
IL1R2	interleukin 1 receptor, type II	−5.2
KLHL1	kelch-like 1 (Drosophila)	−3.9
KITLG	KIT ligand	−1.1
MAP3K5	mitogen-activated protein kinase kinase kinase 5	−1.1
PAX4	paired box 4	−1.3
POLDIP3	polymerase (DNA-directed), delta interacting protein 3	−1.2
PPP2R1B	protein phosphatase 2, regulatory subunit A, beta isoform	−3.5
ROR2	receptor tyrosine kinase-like orphan receptor 2	−1.8
ARHGEF5	Rho guanine nucleotide exchange factor (GEF) 5	−2.7
RBM16	RNA binding motif protein 16	−6.5
SAFB	scaffold attachment factor B	−1.6
SMPD2	sphingomyelin phosphodiesterase 2, neutral membrane	−1.7
TARBP1	TAR (HIV-1) RNA binding protein 1	−2.2
TGFA	transforming growth factor, alpha	−1.2
TUBA1A	tubulin, alpha 1a	−1.8
WDR68	WD repeat domain 68	−1.5

**Figure 4:**
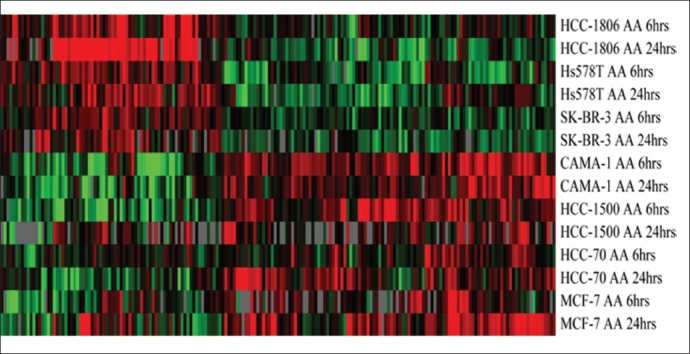
A pseudo color cluster view of genes differentially expressed between ER− and ER+ breast cancer cells in response to AA. Cells were treated with AA at 6 and 24 hrs. RNA was isolated and hybridized on the cDNA microarray slides as detailed in materials and methods. Images were analyzed using GenePix 6.0 and data were analyzed using GeneSpring 10.1

When we used One-way ANOVA with a P-value < 0.05 we identified 437 genes to be differentially expressed between ER− and ER+ breast cancer cells in response to treatment with AA [Figure 4].

Functional annotation of the genes differentially up regulated in ER− and ER+ cells showed that ERK/MAPK, NF-κB, EGF signaling and VEGF signaling cascades were highly enriched in ER+ cells treated with Arachidonic acids while in ER− cells RAR Activation cascade, IL-4 signaling, insulin receptor signaling, and p53 signaling were significantly expressed.

**Figure 5:**
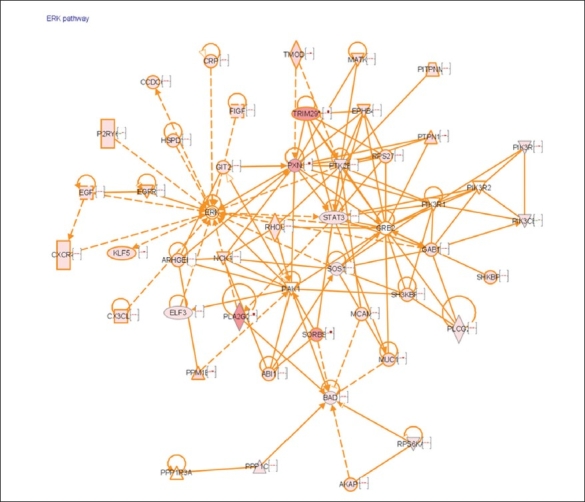
Ingenuity pathway analysis of genes up regulated in ER+ cell in response to AA shows that the ERK/MEK pathway was significantly associated with ER+. Cells were treated with AA at 6 and 24 hrs. RNA was isolated and hybridized on the cDNA microarray slides as detailed in materials and methods. Images were analyzed using GenePix 6.0 and data were analyzed using GeneSpring 10.1

Functional annotation and pathway analysis of genes up regulated mainly in ER+ breast cancer cells in response to Arachidonic acid show that the top ranked pathway was the ERK/MEK signaling pathway [Figure 5]. Table 5 lists the genes from the ERK- pathway that were up regulated by Arachidonic acid in ER+ breast cancer cells.

**Table 5:** ERK pathway related genes up regulated in ER+ breast cancer cells in response to AA

Symbol	Entrez Gene Name	Fold Change
ABI1	abl-interactor 1	2.9
AKAP1	A kinase (PRKA) anchor protein 1	2.6
ARHGEF7	Rho guanine nucleotide exchange factor (GEF) 7	3.0
BAD	BCL2-associated agonist of cell death	2.1
CCDC6	coiled-coil domain containing 6	2.7
CRP	C-reactive protein, pentraxin-related	2.8
CX3CL1	chemokine (C-X3-C motif) ligand 1	2.3
CXCR4	chemokine (C-X-C motif) receptor 4	2.4
EGF	epidermal growth factor (beta-urogastrone)	2.0
EGFR	epidermal growth factor receptor	3.4
ELF3	E74-like factor 3 (ets domain transcription factor, epithelial-specific)	2.4
EPHB4	EPH receptor B4	2.1
FIGF	c-fos induced growth factor (vascular endothelial growth factor D)	2.4
GAB1	GRB2-associated binding protein 1	2.5
GIT2	G protein-coupled receptor kinase interacting ArfGAP 2	2.9
HSPD1	heat shock 60kDa protein 1 (chaperonin)	2.4
KLF5	Kruppel-like factor 5 (intestinal)	3.9
MATK	megakaryocyte-associated tyrosine kinase	2.9
MCAM	melanoma cell adhesion molecule	2.8
MUC1	mucin 1, cell surface associated	3.9
NCK1	NCK adaptor protein 1	3.1
P2RY6	pyrimidinergic receptor P2Y, G-protein coupled, 6	2.7
PIK3CB	phosphoinositide-3-kinase, catalytic, beta polypeptide	2.6
PIK3R3	phosphoinositide-3-kinase, regulatory subunit 3 (gamma)	3.9
PITPNM3	PITPNM family member 3	2.0
PLA2G2A	phospholipase A2, group IIA (platelets, synovial fluid)	12.2
PLCG2	phospholipase C, gamma 2 (phosphatidylinositol-specific)	3.2
PPM1E	protein phosphatase 1E (PP2C domain containing)	4.5
PPP1CA	protein phosphatase 1, catalytic subunit, alpha isoform	2.5
PTK2B	PTK2B protein tyrosine kinase 2 beta	3.0
PTPN12	protein tyrosine phosphatase, non-receptor type 12	4.3
PXN	paxillin	10.8
RHOU	ras homolog gene family, member U	2.3
RPS27A	ribosomal protein S27a	2.7
RPS6KA1	ribosomal protein S6 kinase, 90kDa, polypeptide 1	2.4
SH3KBP1	SH3-domain kinase binding protein 1	2.9
SHKBP1	SH3KBP1 binding protein 1	2.3
SORBS2	sorbin and SH3 domain containing 2	10.9
SOS1	son of sevenless homolog 1 (Drosophila)	3.6
STAT3	signal transducer and activator of transcription 3 (acute-phase response factor)	2.1
TMOD1	tropomodulin 1	5.0
TRIM29	tripartite motif-containing 29	11.9

**Figure 6:**
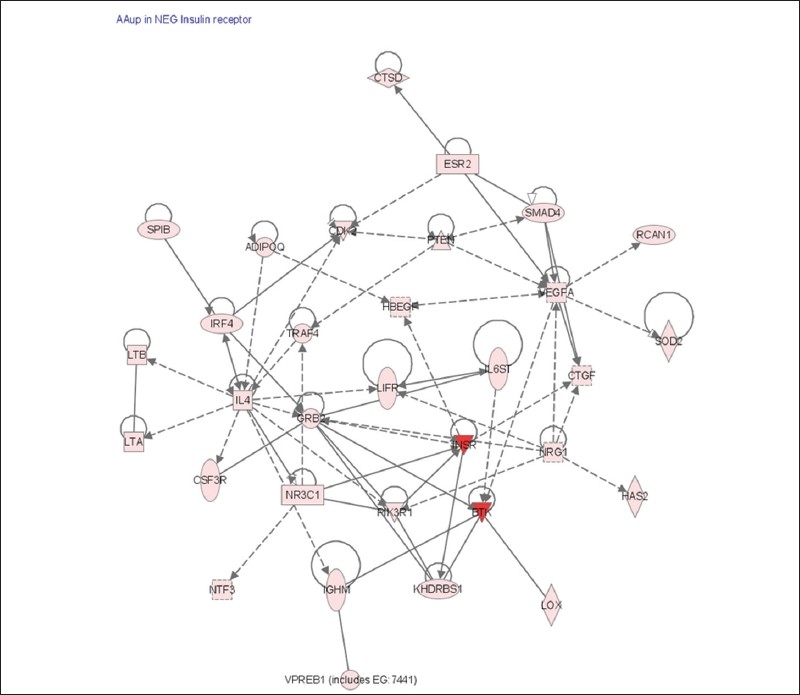
The insulin receptor cascade was uniquely up regulated in ER− cells when treated with AA. Ingenuity pathway analysis of the insulin receptor cascade showing the expression patterns of the pathway component in ER− cells. Cells were treated with AA at 6 and 24 hrs. RNA was isolated and hybridized on the cDNA microarray slides as detailed in materials and methods. Images were analyzed using GenePix 6.0 and data were analyzed using GeneSpring 10.1

We carried out functional annotation for the genes that were up regulated in ER− cells when treated by Arachidonic acid and found that the Insulin Receptor signaling pathway was highly enriched. Vascular endothelial growth factor and superoxide dismutase were also up regulated in ER− cells [Figure 6]. Table 6 lists the insulin receptor pathway genes that were uniquely up regulated in ER− cells.

**Table 6:** Insulin receptor pathway related genes up regulated in ER- breast cancer cells in response to AA

Symbol	Entrez gene name	Fold change
ADIPOQ	adiponectin, C1Q and collagen domain containing	3.4
BTK	Bruton agammaglobulinemia tyrosine kinase	54.9
CDK2	cyclin-dependent kinase 2	2.4
CSF3R	colony stimulating factor 3 receptor (granulocyte)	2.1
CTGF	connective tissue growth factor	2.1
CTSD	cathepsin D	2.2
ESR2	estrogen receptor 2 (ER beta)	2.5
GRB2	growth factor receptor-bound protein 2	2.3
HAS2	hyaluronan synthase 2	2.0
HBEGF	heparin-binding EGF-like growth factor	2.0
IGHM	immunoglobulin heavy constant mu	2.4
IL4	interleukin 4	3.1
IL6ST	interleukin 6 signal transducer (gp130, oncostatin M receptor)	2.5
INSR	insulin receptor	43.5
IRF4	interferon regulatory factor 4	2.1
KHDRBS1	KH domain containing, RNA binding, signal transduction associated 1	4.6
LIFR	leukemia inhibitory factor receptor alpha	2.5
LOX	lysyl oxidase	2.3
LTA	lymphotoxin alpha (TNF superfamily, member 1)	2.0
LTB	lymphotoxin beta (TNF superfamily, member 3)	2.0
NR3C1	nuclear receptor subfamily 3, group C, member 1 (glucocorticoid receptor)	2.1
NRG1	neuregulin 1	4.5
NTF3	neurotrophin 3	2.5
PIK3R1	phosphoinositide-3-kinase, regulatory subunit 1 (alpha)	3.5
PTEN	phosphatase and tensin homolog	2.3
RCAN1	regulator of calcineurin 1	2.9
SMAD4	SMAD family member 4	3.7
SOD2	superoxide dismutase 2, mitochondrial	3.8
SPIB	Spi-B transcription factor (Spi-1/PU.1 related)	2.4
TRAF4	TNF receptor-associated factor 4	2.2
VEGFA	vascular endothelial growth factor A	2.5
VPREB1	pre-B lymphocyte 1	4.5

**Figure 7:**
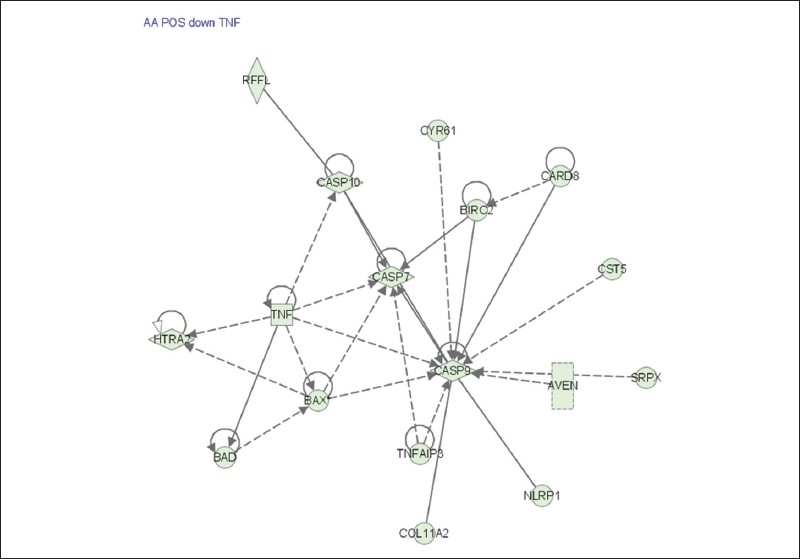
Pathway analysis of genes involved in cell-cell signaling significantly associated with ERbreast cancer cells in response to AA. This cascade was down regulated in ER− cells only. Cells were treated with AA at 6 and 24 hrs. RNA was isolated and hybridized on the cDNA microarray slides as detailed in materials and methods. Images were analyzed using GenePix 6.0 and data were analyzed using GeneSpring 10.1

Of the genes that were down regulated by AA uniquely in ER+ cells are genes involved in apoptosis such as Caspase 7, Caspase 9, Caspase 10, TNF, and BCL-2 associated agonist of cell death [Figure 7].

The peroxisome proliferator-activated receptor (PPAR) related pathways were significantly down regulated in ER+ cells by Arachidonic acid. These genes are listed in Table 7.

**Table 7:** Peroxisome proliferator-activated receptor (PPAR) pathwaysrelated genes down regulated in ER+ breast cancer cells in Response to AA

Symbol	Entrez gene name	Fold change
ADCY10	adenylate cyclase 10 (soluble)	−3.7
ASPN	asporin	−8.8
CD36	CD36 molecule (thrombospondin receptor)	−3.9
CYP2C19	cytochrome P450, family 2, subfamily C, polypeptide 19	−9.6
CYP2C8	cytochrome P450, family 2, subfamily C, polypeptide 8	−2.4
CYP2C9	cytochrome P450, family 2, subfamily C, polypeptide 9	−5.9
FASN	fatty acid synthase	−2.6
GH1	growth hormone 1	−2.9
GHR	growth hormone receptor	−21.4
GNAS	GNAS complex locus	−15.1
HRAS	v-Ha-ras Harvey rat sarcoma viral oncogene homolog	−2.4
IKBKB	inhibitor of kappa light polypeptide gene enhancer in B-cells, kinase beta	−2.3
IL1R2	interleukin 1 receptor, type II	−4.1
IL6	interleukin 6 (interferon, beta 2)	−11.8
KRAS	v-Ki-ras2 Kirsten rat sarcoma viral oncogene homolog	−2.3
MAP2K3	mitogen-activated protein kinase kinase 3	−3.7
MAP3K7	mitogen-activated protein kinase kinase kinase 7	−2.3
MED1	mediator complex subunit 1	−2.3
NCOA3	nuclear receptor coactivator 3	−2.4
NFKBIE	nuclear factor of kappa light polypeptide gene enhancer in B-cells inhibitor, epsilon	−3.8
PLCE1	phospholipase C, epsilon 1	−2.9
PLCL1	phospholipase C-like 1	−31.2
PRKACA	protein kinase, cAMP-dependent, catalytic, alpha	−10.5
PRKACB	protein kinase, cAMP-dependent, catalytic, beta	−4.9
PRKAR2B	protein kinase, cAMP-dependent, regulatory, type II, beta	−3.2
REL	v-rel reticuloendotheliosis viral oncogene homolog (avian)	−14.7
RELA	v-rel reticuloendotheliosis viral oncogene homolog A (avian)	−13.5
RRAS	related RAS viral (r-ras) oncogene homolog	−2.3
SMAD4	SMAD family member 4	−3.6

**Figure 8:**
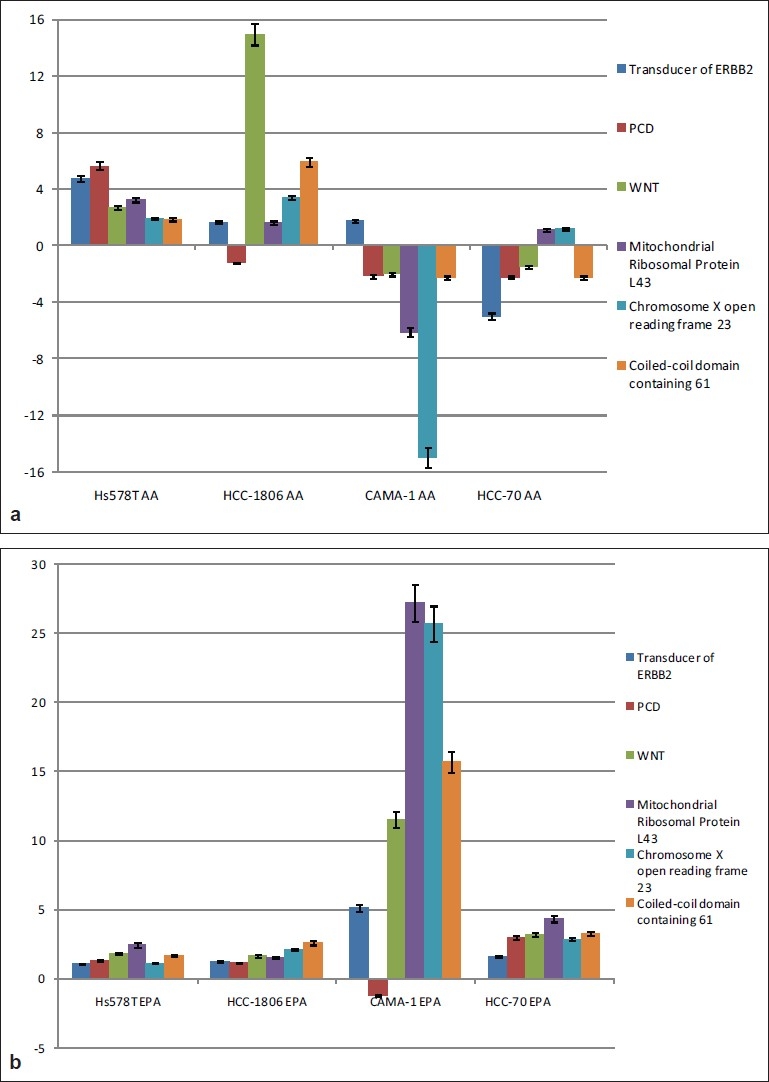
The gene expression data obtained by Real-time PCR experiment. ER− cells (HCC-1806 and Hs578T) and ER+ cells (CAMA-1 and HCC-70) were incubated with either AA (A.) or EPA (B.) for six hours. At the end of the incubation period, the cells were washed with PBS and TriZol was added. Total RNA was isolated analyzed using RT-PCR. Data points are the mean and standard error of three independent experiments for the same samples used in the microarray experiments. Gene expression data were normalized to GAPDH mRNA that showed no regulation among the various treatments compared to the untreated control cells

Five genes that were regulated were selected for real-time polymerase chain reaction (PCR). These genes are *Protocadherin, thyroid hormone receptor-associated protein complex component (TRAP150), Mitochondrial ribosomal protein L43, transducer of ERBB2, WNT-2B Isoform 1 oncogene and coiled-coil domain containing 61 (CCDC61).* Real-time PCR was carried out using samples from ER− cells (HCC-1806 and Hs578T) and ER+ cells (CAMA-1 and HCC-70) treated with the fatty acids and compared to the control untreated cells [Figure 8].

## DISCUSSION

Previous studies have emphasized that the correlation between postmenopausal breast cancer risk and dietary consumption is, for the most part, dependent upon the estrogen receptor status. Scientists have reported that the association between the Alternate Healthy Eating Index (AHEI), the Recommended Food Score (RFS) and the risk of breast cancer were found only in ER− tumors.[12]

Many studies focused on the effect of dietary intake of fatty acids and other nutrient on breast cancer. However, a detailed understanding of the correlation between dietary fat intakes and the ER status of breast cancer is not very well achieved.

A study on the association of alcohol intake and breast cancer risk showed no association between alcohol intake and the risk of developing ER− tumors while a statistically significant correlation between alcohol intake and the risk of developing ER+ tumors was observed.[13] McCann *et al.* have shown that the anti-tumor effects of dietary lignans, found in flaxseed, sesame seed and oat bran, are limited to ER− breast tumors.[14]

A study of the dietary intake of fatty acids in premenopausal breast cancer patients found an association between linoleic acid intake and a higher risk of ER− than ER+ breast tumors.[5] The omega-3 fatty acids, EPA and DHA, are shown to inhibit the growth of ER− and ER+ breast cancer cells *in vitro*.[15,16]

In this study, we determine whether the ER status of breast cancer cells plays a role in their responses to fatty acids at the molecular level, using microarrays. We have identified genes and pathways that are differentially expressed between ER− and ER+ cells in response to EPA and AA. The effect of EPA on cell-cell signaling was dependent on the ER status and included the activation of the caspase cascade in ER+ cells while the activation of the Cyclin-dependent kinase cascade was uniquely activated in ER− cells.

Functional interactions between ER and beta-catenin through transcriptional modulation is an important factor for *in vivo* cross-talk of beta-catenin and estrogen signaling pathways. Transcription coactivators and chromatin remodeling complexes that are normally recruited by beta-catenin are shown to interact with ER, and yet ER and beta-catenin are reciprocally recruited to cognate response elements in the promoters of their target genes. This interaction may underlie the pathological conditions in which abnormalities of beta-catenin signaling have been implicated.[17] In tumor cells, expression of ER down regulates beta-catenin and its target genes, cyclin D1 and Rb, important regulators of cell cycle and cell proliferation. Over expression of ER induces cellular apoptosis by inducing hTNF-alpha gene expression, which in turn activates caspases -8, -9 and -3 and lead to DNA fragmentation.[18]

One of the pathways that were differentially expressed in ER+ cells in response to AA was the ERK/MEK pathway.

For many years, the involvement of the ERK/MEK cascade in cell growth and the prevention of apoptosis have been investigated. Studies have shown that the ERK/MEK pathway can induce the progression of cancer cells due in part to the inhibition of apoptosis.[19–22]

It has been well documented that there is cross talk between the ER pathway and the ERK/MEK cascade and that the ERK/MEK pathway is regulated by estrogen in ER+ cells in a Ca+2-dependent manner, and that the anti-apoptotic effect of estrogen may be partly dependent on the ERK/MEK pathway.[23,24]

Arachidonic acid is shown to differentially induce the insulin signaling pathway in ER− cells. Genes involved in insulin receptor signaling pathway such as insulin like growth factors (IGF-I and –II) have been found to induce growth of many breast cancer cells. Expression of IGF-I receptor (IGF-IR) shown to be highly activated in breast tumors in comparison with normal epithelial cells.[25] Over expression of insulin receptor signaling genes, which aggravate proliferation of breast cancer cells, is worse in ER− patients. For example, ER− breast cancer patients have higher insulin like growth factor binding proteins levels than ER+ patients.[26] Elevated expression of IGF-IR or Insulin receptor substrate 1 (IRS-1) appears to increase drug- and radio-resistance of breast cancer cells and favor cancer recurrence.[25,27] Insulin receptor substrate 1 (IRS-1) is important in transmitting IGF-IR signals to counteract ER apoptotic effect through the PI-3K/Akt survival pathways, and its stabilization improved survival of breast cancer cells in the presence of IGF-I.[28]

Also documented is the cross-talk between the PPAR and ER pathways has been documented.[29,30] The PPAR cascade was uniquely down regulated in ER+ cells in response to arachidonic acid and not altered in ER− cells.

Our findings suggest that the ER status of breast cancer cells may play a role in breast cancer cell response to treatments with omega-3 and omega-6 fatty acids.

Further investigation of these pathways may shed light on the importance of the ER status on the mechanistic and therapeutic/preventive roles of fatty acids in breast cancer.

## AUTHOR'S PROFILE

**Dr. Alquobaili** is a professor in the Department of Biochemistry and Microbiology at the Damascus University School of Pharmacy, Damascus, Syria.

**Dr. Seid Muhie**, got his PhD from Georgetown in 2007. He is Post Doctoral fellow in Dr. Marti Jett's Lab at Walter Reed Army Institute of Research. He is working on bioinformatics analyses of high throughput data and systems biology of immune response to different pathogens and malignancy.

**Dr. Agnes A. Day** is the Chairman of the Department of Microbiology in the Howard University College of Medicine, and the Research Director of the Laboratory of Molecular Metastasis in the Howard University Cancer Center. Her research interests include development of drug resistance in pathogenic dimorphic fungi, the roles of connective tissue proteins in cellular growth and cancer metastasis and the molecular differences in breast cancer occurrence between ethnic groups. The development of animal models of metastatic breast cancer, the role of dietary components in breast cancer induction and the molecular characterization of the aggressive phenotype of breast cancer in African American women are the current foci of her research. Results of these studies have identified differential expression of specific matrix metalloproteinases and other cancer associated genes between breast cancer cells derived from Caucasian and African American women.

**Dr. Rasha Hammamieh** is a senior scientist at the Department of Molecular Pathology, the Walter Reed Army Institute of Research. She is involved in genomic study of cellular responses to stressors and in identifying and analyzing the biochemical pathways and networks in response to various treatments. She is also involved in studying the effects of Omega-3 and Omega-6 fatty acids on cancer cells.

**Stacy-Ann Miller** holds a degrees in Biological Sciences and Romance Languages (French and Italian) from the University of Maryland College Park. She is an associate researcher and the lab manager at the Walter Reed Army Institute of Research at the Department of Molecular Pathology where she was involved with genomics research in the fields of breast cancer, post-traumatic stress disorder, and host gene expression responses to bio threat agents. Her research interests include genomics, cancer biology, bioinformatics, basic and preclinical research in infectious diseases host immune responses.

**Dr. Marti Jett** is Chief, Department of Molecular Pathology at WRAIR. She has studied mechanisms of action of biothreat pathogenic agents for ∼15 years. Her laboratory is presently focused on establishing a library of host gene expression responses to biological threat agents. This work started with seed funding in which the question being addressed was to determine differences and similarities in gene expression profiles between two shockinducing toxins that result in similar progression of lethal illness. This work provided the preliminary evidence for a major research funding initiative award from several national biomedical institutes. The overall aim of these studies is to identify host gene profiles related to progression of illness and integrated with clinical observations. The networks and regulatory nodes that emerge from these studies have the potential to aid in understanding the “course of impending illness”, determining the status of individuals with stage-specific identification of illness progression and can provide therapeutic approaches as these illnesses progress upon exposure to these intractable illnesses.
